# Palbociclib inhibits epithelial-mesenchymal transition and metastasis in breast cancer via c-Jun/COX-2 signaling pathway

**DOI:** 10.18632/oncotarget.5993

**Published:** 2015-10-19

**Authors:** Ge Qin, Fei Xu, Tao Qin, Qiufan Zheng, Dingbo Shi, Wen Xia, Yun Tian, Yanlai Tang, Jingshu Wang, Xiangshen Xiao, Wuguo Deng, Shusen Wang

**Affiliations:** ^1^ Sun Yat-Sen University Cancer Center, State Key Laboratory of Oncology in South China, Collaborative Innovation Center of Cancer Medicine, Guangzhou 510060, China; ^2^ Department of Pediatrics, The First Affiliated Hospital, Sun Yat-Sen University, Guangzhou 510080, China

**Keywords:** palbociclib, breast cancer, metastasis, epithelial-mesenchymal transition, COX-2

## Abstract

Palbociclib, a highly selective CDK4/6 inhibitor, has been shown to be a novel anti-tumor agent that suppresses breast cancer cell proliferation. However, its anti-metastasis activity remains controversial. In the present study, we evaluated whether palbociclib prevented breast cancer cell metastasis and revealed its regulatory mechanism. We found that palbociclib inhibited migration and invasion in the breast cancer cells MDA-MB-231 and T47D. The epithelial-mesenchymal transition (EMT) markers, vimentin and Snail, were down-regulated with palbociclib treatment. Moreover, we revealed that this inhibition was mediated by the c-Jun/COX-2 pathway. COX-2 was decreased after palbociclib treatment. The production of PGE2 was also reduced along with COX-2. Additionally, our data showed that c-Jun, a crucial transcriptional regulator of COX-2, was down-regulated by palbociclib. We found that palbociclib weakened the COX-2 promoter binding activity of c-Jun and prevented its translocation from the cytoplasm to cell nuclei. Bioluminescence imaging and tail intravenous injection were used to evaluate the anti-metastasis effect of palbociclib *in vivo*. The data demonstrated that palbociclib reduced breast cancer metastasis to the lung. These results therefore demonstrated that the anti-metastasis activity of palbociclib is mediated via the c-Jun/COX-2 signaling pathway by inhibiting EMT in breast cancer cells.

## INTRODUCTION

Breast cancer is a common malignancy and a significant cause of death in females worldwide [1, 2]. Encouraging progress has been made with regard to the diagnosis and treatment of breast cancer in recent years. Chemotherapy, endocrine therapy and targeted agents have remarkably improved the overall survival (OS) and disease-free survival (DFS) rates of breast cancer [3–5]. However, metastasis and recurrence are still the critical clinical events in breast cancer. Even in node-negative breast cancer patients, 25% of patients develop metastasis [6]. The 5-year survival rate is dramatically lowered among patients with distant metastasis. Therefore, extensive efforts are required to explore novel therapeutic targets to control metastasis and improve the quality of life among breast cancer patients.

Palbociclib (PD0332991), a potent and highly selective CDK4/6 inhibitor, has raised great concern these years. This small molecule compound can block the cell cycle in the G0/S1 phase, which is a key checkpoint in cell cycle regulation. Thus, this attribute is considered to have the potential to suppress tumor progression [7]. In practical applications, Palbociclib has shown effectiveness in the treatment of different types of tumors, especially breast cancer. Richard S. Finn et al. revealed that Palbociclib could inhibit the growth of multiple breast cancer cell lines that were at a relatively low density, particularly ER-positive cells [8]. *In vivo*, Palbociclib exerts its anti-tumor activity by inhibiting the growth of human tumor xenografts and down-regulating the level of the proliferation marker Ki67 [9]. This CDK4/6 inhibitor has mostly been reported to be a powerful cytostatic agent in ER-positive breast cancer and to have a synergistic effect when combined with anti-hormonal agents. There are numerous preclinical data regarding the synergy between Palbociclib and anti-estrogen agents. [8, 10] PALOMA-1/TRIO-18, an open-label, randomized phase 2 clinical trial confirmed that the addition of Palbociclib to letrozole improved the PFS of postmenopausal women with advanced ER-positive breast cancer for 18.1 months [11]. The growth-inhibitory activity of Palbociclib in breast cancer cells is mediated by the CDK4/6-Rb-E2F axis [12]. Palbociclib inhibits the phosphorylation of Rb and then influences the transcription activity of the E2F transcription factor family, which have a large number of target genes that regulate cell proliferation and apoptosis [13]. Although it had been shown that Palbociclib can suppress breast cancer proliferation, its anti-metastasis activity is still unclear. Moreover, the therapeutic potential of palbociclib in ER-negative breast cancer is controversial.

Breast cancer is a malignant tumor with a strong tendency to metastasize, and the epithelial-mesenchymal transition process (EMT) is considered to play a vital role in cancer metastasis [14]. During the EMT process, cells lose epithelial characteristics and obtain a mesenchymal phenotype, which weakens the cell-cell adhesion and improves cell motility [15]. EMT is based on the down-regulation of epithelial markers such as E-cadherin, occludins and claudins while the mesenchymal markers, such as vimentin and N-cadherin, are up-regulated. Moreover, EMT-inducing transcriptional factors, such as Snail, Slug, Twist and FOX2, are affected through EMT progression [15, 16]. Cyclooxygenase-2 (COX-2), an inflammation-related enzyme, was shown to be linked to EMT and, therefore, critical for breast cancer motility, invasion and metastasis [17]. COX-2 is over-expressed in many malignant human cancers and is associated with poor prognosis [18, 19]. The enzyme was found to play a key role in the initiation of various processes, including apoptosis, angiogenesis and metastasis. COX-2-induced PGE2 production was reported to assist migration and EMT progress in human breast cancer cells [20]. Moreover, *in vivo* studies further indicated that specific inhibition or knockout of COX-2 reduced cancer metastasis in mouse models [21].

In this study, we hypothesized that Palbociclib would inhibit the invasion and metastasis of breast cancer cells and down-regulate the expression of EMT markers through the modulation of the COX-2 pathway. Evidence is provided that Palbociclib decreases COX-2 expression, PGE2 abundance and vimentin and Snail expression. Furthermore, the transcriptional regulation of COX-2 was evaluated using pull-down and immunofluorescence assays. Additionally, the anti-metastasis activity of Palbociclib was verified by establishing a spontaneous metastatic model in nude mice.

## RESULTS

### Palbociclib decreased Rb phosphorylation

**Figure 1:**
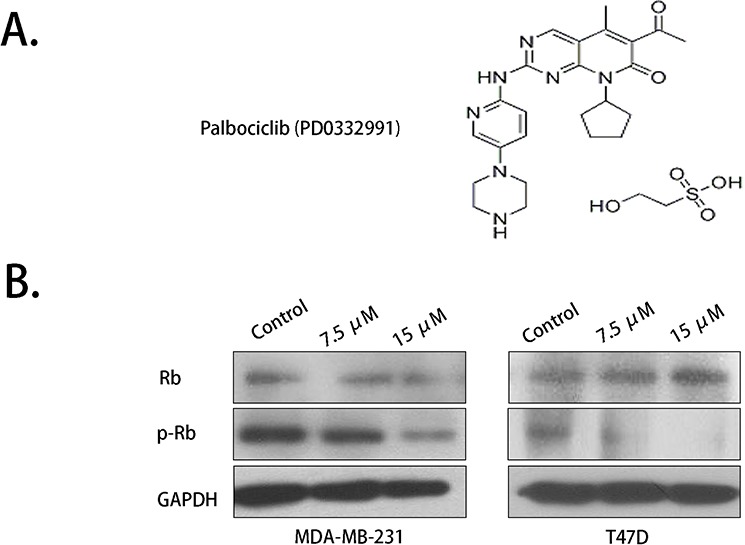
Rb phosphorylation was inhibited by palbociclib **A.** The molecular structure of palbociclib. **B.** Total Rb expression showed no significant variation while phosphorylated Rb (Ser 780 site) was down-regulated by palbociclib.

The molecular structure of palbociclib was obtained from Pfizer's product manual and was showed in Fig. 1A. Rb was proved to be a key target of palbociclib. Rb deficiency resulted in primary resistant of palbociclib. Western blot was conducted to detect Rb expression in MDA-MB-231 and T47D breast cancer cells. The results showed that Rb phosphorylation (Ser 780) was inhibited by palbociclib in a concentration-dependent manner (Fig. 1B). However, no significant proliferation inhibition activity was detected by MTS assay in reasonable concentrations of the drug. (Supplementary Fig. A).

### Palbociclib inhibited breast cancer cell migration and invasion and down-regulated the expression of EMT markers

**Figure 2:**
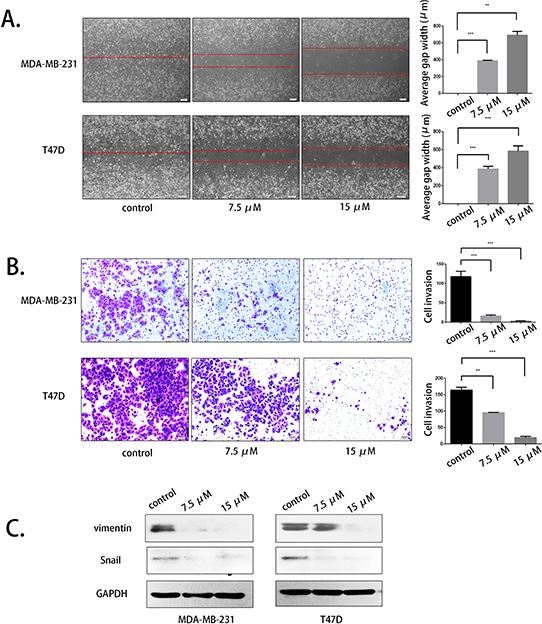
Palbociclib inhibited breast cancer cell migration and invasion by suppressing vimentin and Snail **A.** MDA-MB-231 and T47D cells were treated with DMSO (control) or 7.5 μM or 15 μM palbociclib for 48 h after scratching. The average gap width was used to evaluate migration. **B.** MDA-MB-231 and T47D cells were treated with the indicated concentration of palbociclib for 48 h, and the transwell assay was applied to assess invasion. The number of invading cells was calculated in three different fields. **C.** The cells were treated with the same conditions as before. The expression of vimentin and Snail was determined by western blot.

To evaluate the effect of palbociclib on migration, we first used the scratch assay. Palbociclib was used at 7.5 μM and 15 μM. After observation for 48 hours, we found that the migration abilities of MDA-MB-231 cells and T47D cells were inhibited in a concentration-dependent manner (Fig. 2A). The wounding space of the control groups was 100% occupied by migrating cells after 48 hours, which contrasted with the relatively wider gap of the palbociclib-treated groups. However, the cell density and morphology of the cells were not significantly different in the treatment groups.

We next analyzed the effect of palbociclib on invasion by conducting transwell assays. After 16–18 hours, palbociclib was shown to inhibit the invasion of both MDA-MB-231 and T47D cells in a dose-dependent manner. The number of invading cells in the high-concentration group was tens times less than that of untreated group (Fig. 2B). The number of invasive cells was calculated in three different fields.

Vimentin and Snail are two crucial molecules involved in EMT progression and have a vital role in breast cancer cell migration and invasion. We therefore analyzed the effects of palbociclib on the expression of these two EMT markers by western blot. As expected, the drug-treated cells had a lower expression of vimentin and Snail, especially at the higher concentration of 15 μM (Fig. 2C).

### Palbociclib inhibited COX-2 expression and PGE2 secretion

**Figure 3:**
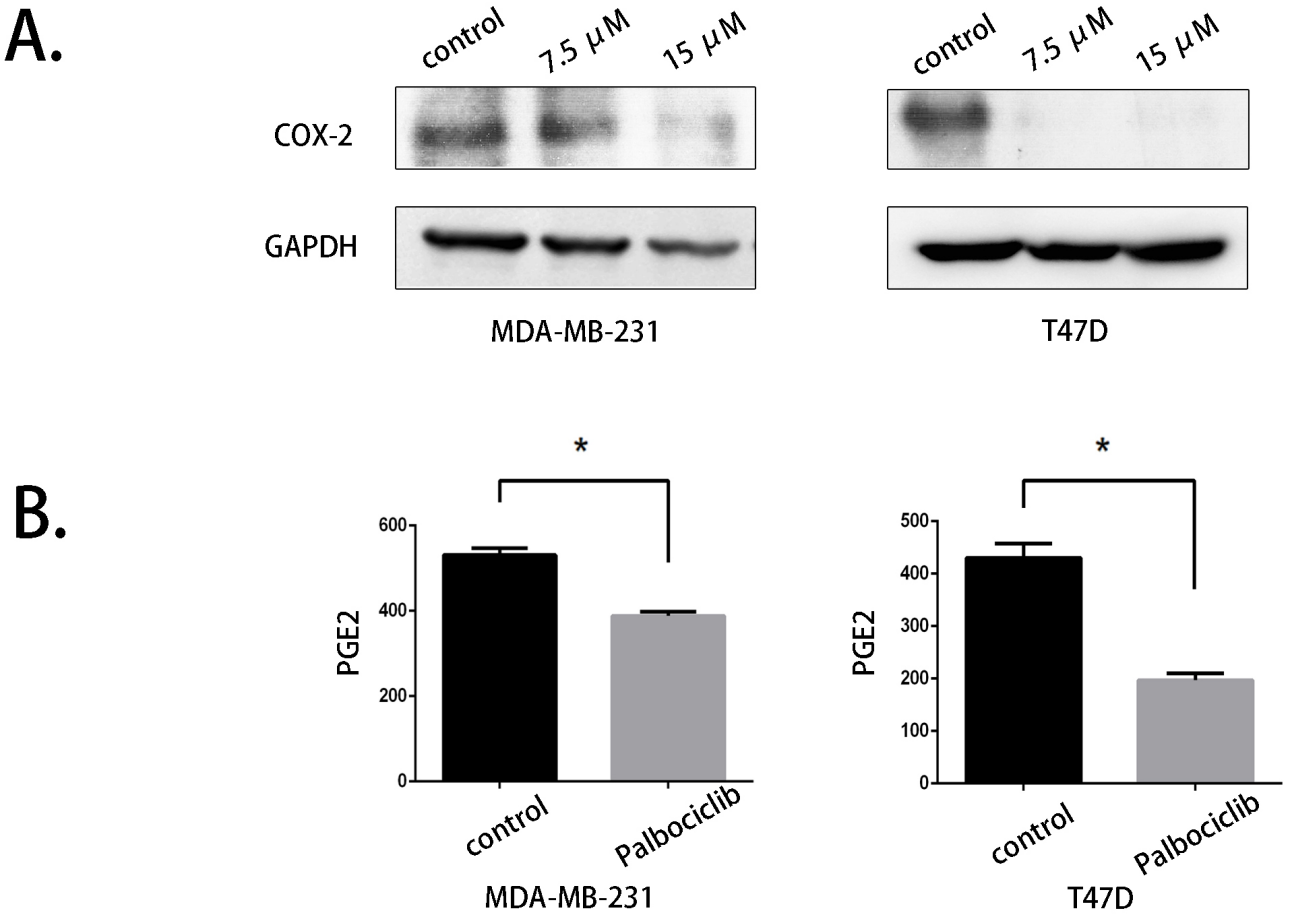
COX-2 expression and PGE2 production were reduced by palbociclib **A.** COX-2 expression was evaluated by western blot after palbociclib (0 μM, 7.5 μM, 15 μM) treatment for 48 hours. **B.** The supernatant of control and 7.5 μM palbociclib-treated cells was collected and stored at −80°C. PGE2 secretion was detected using ELISA.

COX-2 was shown to be up-regulated in breast cancer and a novel metastasis-related factor. The migration- and invasion-promoting effects of COX-2 were mediated by the induction of PGE2 secretion. PGE2 then increased the expression of EMT markers through multiple pathways. We evaluated the protein expression level of COX-2 using western blot assays, and the results confirmed that palbociclib significantly reduced COX-2 expression at concentrations of 7.5 μM and 15 μM (Fig. 3A).

We performed further studies to determine whether palbociclib could down-regulate PGE2 secretion. We collected the culture medium of control cells and palbociclib-treated cells (7.5 μM) and detected PGE2 levels using ELISA. As shown in Fig. 3B, The PGE2 level in palbociclib-treated cells was dramatically lower than in untreated cells, which was consistent with the decrease of COX-2 expression.

### The selective COX-2 enzymatic inhibitor celecoxib suppressed breast cancer cell migration and invasion

**Figure 4:**
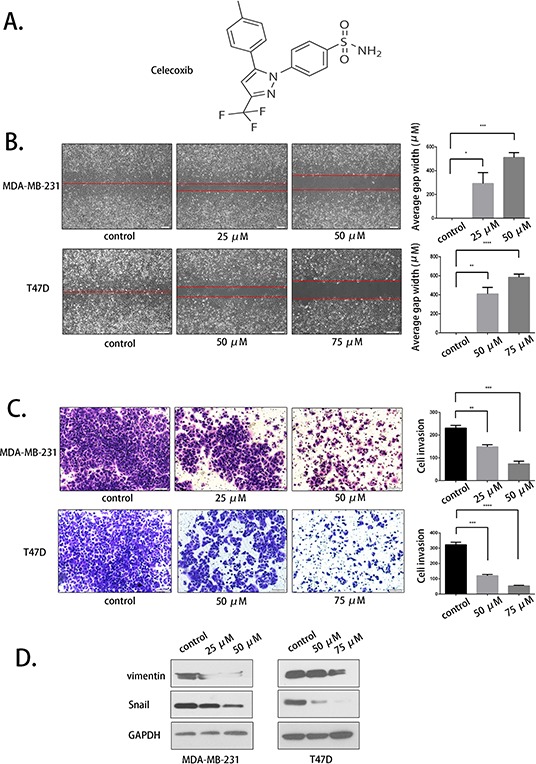
Selective COX-2 inhibition by celecoxib suppressed breast cancer cell migration and invasion **A.** The molecular structure of celecoxib. **B.** The COX-2 inhibitor celecoxib suppressed the migration of MDA-MB-231 and T47D cells at the indicated concentrations. **C.** The invasion-inhibiting effect of celecoxib. **D.** Vimentin and Snail expression was evaluated after celecoxib treatment.

Because COX-2 has been reported to have a crucial role in breast cancer metastasis, we used celecoxib, a selective COX-2 inhibitor, to evaluate its activity. The molecular structure of celecoxib was obtained from Sigma's product manual (Fig. 4A). Scratch and transwell assays were conducted to evaluate celecoxib at concentrations of 25 μM and 50 μM in MDA-MB-231 and 50 μM and 75 μM in T47D cells for 48 hours. The results demonstrated that celecoxib inhibited the migration and invasion of the two cell lines in a dose-dependent manner (Fig. 4B and Fig. 4C). Western blot assays were used to evaluate the expression of vimentin and Snail again, and these EMT markers were down-regulated with celecoxib treatment (Fig. 4D). These data indicate that COX-2 inhibition was indeed related to breast cancer cell migration and invasion.

### Celecoxib blocked while LPS partly overcame the anti-metastastasis activity of palbociclib

**Figure 5:**
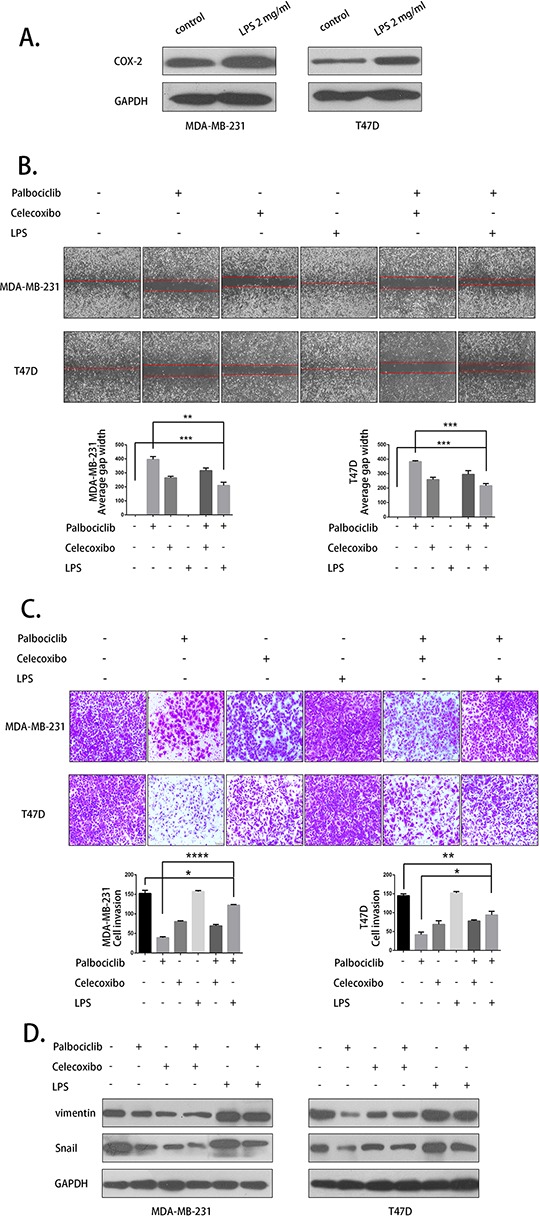
LPS partly rescued and celecoxib weakend the anti-metastasis activity of palbociclib **A.** LPS up-regulated COX-2 expression. **B.** The migration assay was conducted with 25 μM celecoxib or 2mg/ml LPS pretreatment and 0 μM or 7.5 μM palbociclib treatment for 48 h. **C.** The invasion assay was conducted using the aforementioned treatment. **D.** Vimentin and Snail expression was detected by western blot.

Palbociclib activity was then further evaluated with cells that were pretreated with celecoxib and LPS. LPS was used to active COX-2 (Fig. 5A). Celecoxib (25 μM), LPS (2mg/ml) and palbociclib (7.5 μM) were used in the migration and invasion assay. As shown in Fig. 5B and Fig. 5C, the migration and invasion inhibition effect of palbociclib was weakened by celecoxib pre-treatment. In addition, this inhibition activity was partly rescued by LPS. The expression of vimentin and Snail was detected by western blot and the result was consistent to migtation and invasion assay (Fig. 5D). Our data indicated that COX-2 plays a key role in the anti-metastasis activity of palbociclib.

### Palbociclib down-regulated the expression of the transcription factor c-Jun

**Figure 6:**
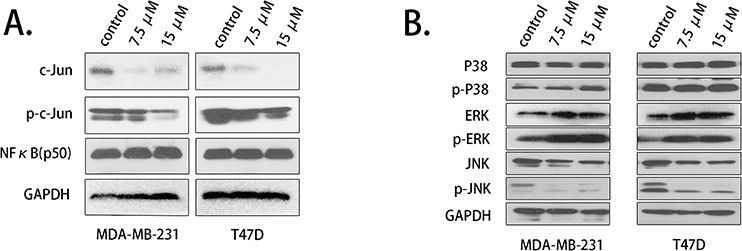
Palbociclib down-regulated c-Jun and JNK expression **A.** The expression of c-Jun and phosphorylated c-Jun was assessed using western blot after the cells were treated with palbociclib. **B.** MAPK pathway-related proteins, p38, ERK, JNK expression levels were detected.

To further evaluated the transcriptional regulation mechanisms of COX-2, we first studied its two important transcription factors, NFκB and c-Jun. The expression level of NFκB (p50) and c-Jun was detected by western blot. c-Jun expression was down-regulated by 7.5 μM and 15 μM palbociclib, while the protein level of NFκB showed no significant differences. We next determined the expression of phosphorylated c-Jun (*p*-*c*-Jun), which is the activated form of c-Jun. The results showed that *p*-*c*-Jun expression was decreased in treated cells, similar to total c-Jun (Fig. 6A).

c-Jun is mainly activated by MAPK pathway. We detected the upstream regulator of c-Jun and found that c-Jun N-terminal kinase (JNK), a critical activator of c-Jun, was down-regulated by palbociclib treatment, while p38 showed no decrease (Fig. 6B). ERK and *p*-ERK up-regulation may leading by the feedback mechanism (Supplementary Fig. B).

### Palbociclib weakened the COX-2 promoter-binding activity of c-Jun and prevented its translocation from the cytoplasm to the nucleus

**Figure 7:**
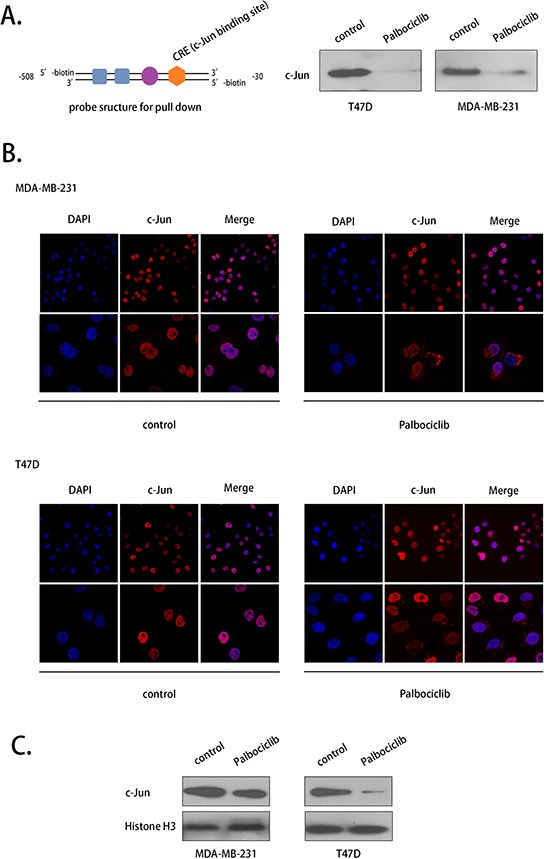
Palbociclib affected the COX-2 promoter-binding activity and the translocation of c-Jun **A.** A pull-down assay was conducted to evaluate the DNA binding activity of c-Jun using a specific probe after drug treatment. C-Jun binding to the probe was washed off and detected by western blot. The structure of the probe is shown. **B.** c-Jun translocation was evaluated using immunofluorescence. The cells were treated with palbociclib (7.5 μM) or DMSO (control) for 12 h. **C.** c-Jun expression in nuclear fraction was evalutaed.

To explore the regulation mechanism between c-Jun and COX-2, we used pull-down and immunofluorescence assays to assess COX-2 DNA binding activity and the translocation of c-Jun. c-Jun was shown to bind to the promoter region of COX-2, and the binding activity was weakened by palbociclib at 7.5 μM (Fig. 7A).

As a transcription factor, c-Jun promotes the transcription of COX-2 when it is located in the nucleus. An immunofluorescence assay indicated that c-Jun was primarily located in the nuclei in MDA-MB-231 and T47D cell lines in the absence of palbociclib. After treatment with palbociclib (7.5 μM) for 12 hours, however, c-Jun appeared in the cytoplasm in some of the cells (Fig. 7B). c-Jun expression was consistently decreased with palbociclib treated in nuclear fraction (Fig. 7C). These data indicate that palbociclib prevented the entry of c-Jun to the nucleus to promote COX-2 transcription.

### Palbociclib reduced the lung metastasis of MDA-MB-231-Luc cells in animal models and decreased COX-2 and c-Jun expression in tumor tissues

**Figure 8:**
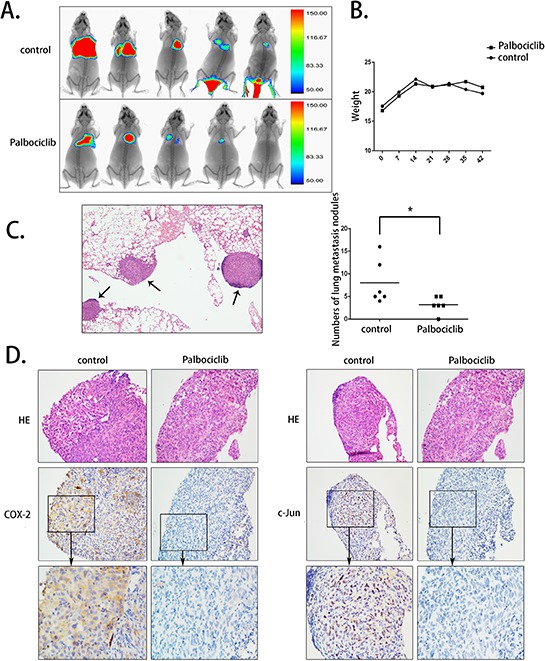
The anti-metastasis activity of palbociclib *in vivo* **A.** The lung metastases of the nude mice are shown using bioluminescence imaging. **B.** The average weight of the two groups. **C.** The HE stain of the lung tissue and the metastasis tumors is noted. The amount of the metastasis tumor was calculated under microscopy. **D.** The expression levels of COX-2 and c-Jun in tumor tissue were assayed by immunohistochemical staining.

An *in vivo* study was conducted to further evaluate the anti-metastasis effect of palbociclib. A lung metastasis animal model was generated through tail vein injections. The MDA-MB-231 cell line was selected for its strong metastasis tendency and stably transfected with luciferase. Bioluminescence imaging (BLI) was applied to visualize lung metastases, and the mice were imaged once every week starting 3 weeks after cell injection. The final BLI data showed that the signal in untreated mice was substantially stronger than that in palbociclib-treated mice using the same BLI parameters, which indicates that the tumor burden was alleviated by palbociclib (Fig. 8A). The weight of each mouse was also measured weekly; the average weights of the two groups showed no significant differences (Fig. 8B).

HE staining was used to confirm pathological metastasis in the lungs, and the metastatic tumors were calculated by microscopy. In accordance with BLI observation, the amount of pulmonary metastatic tumors in vehicle-treated mice ranged from 4 to 16, while that in palbociclib-treated mice ranged from 0 to 5 (Fig. 8C).

COX-2 and c-Jun expression was shown to decrease *in vitro* after palbociclib treatment. To determine their expression in metastatic tumor tissue, immumohistochemical staining was conducted. As shown in Fig. 8D, although COX-2 expression was not apparently high, as expected, its expression was down-regulated in palbociclib-treated mice. The staining of c-Jun was consistent with that of COX-2; c-Jun expression was significantly high in vehicle-treated mice but was almost negative in palbociclib-treated mice. These data indicate that palbociclib prevented MDA-MB-231-Luc cells from metastasizing and decreased the expression of COX-2 and c-Jun *in vivo*.

## DISCUSSION

In this study, we analyzed the anti-metastasis effect of palbociclib (PD0332991) on breast cancer cells. We found that palbociclib significantly suppressed cell migration and invasion. The expression of vimentin and Snail was simultaneously down-regulated. Our results also showed that palbociclib inhibited breast cancer cell migration and invasion by decreasing COX-2 expression and PGE2 production. Celecoxib, a selected inhibitor of COX-2, was shown to suppress breast cancer cell motility and invasiveness. When combined with LPS, the migration-and invasion-inhibition activities of palbociclib were partly rescued. Moreover, we found that palbociclib decreased COX-2 expression by mediating c-Jun, an important transcription factor of COX-2. An *in vivo* study demonstrated that palbociclib accelerated lung metastasis, which was consisted with our *in vitro* study.

The proliferative suppression of breast cancer by CDK4/6 inhibition has been shown by a number of pre-clinical studies [7, 8]. Palbociclib was reported to arrest the cell cycle at G1/S phase by suppressing retinoblastoma (Rb) protein phosphorylation [12, 24]. Rb protein exerts its activity by interacting with the E2F transcription factor family [25, 26]. However, its effect on breast cancer metastasis has not yet been clearly demonstrated. Rebecca Lamb et al. revealed that palbociclib exerted divergent functions in breast cancer stem cell-like activity depending on estrogen receptor (ER) status. Palbociclib suppressed mammosphere formation only in ER-positive cells [27]. In contrast, other research showed that inhibiting CDK4/6 activity decreased migration and invasion activity in triple-negative cell lines [28]. Because of these controversial reports, we evaluated the anti-metastasis property of palbociclib in two molecular subtypes of breast cancer cells. Our results showed that palbociclib inhibited breast cancer cell migration and invasion both in ER-positive (T47D) and -negative (MDA-MB-231) cell lines. The data indicated that Palbociclib may exert its anti-metastasis activity through similar regulatory mechanisms in different breast cancer cell lines.

The research on the mechanisms of palbociclib had primarily focused on Rb function. Our data also showed Rb phosphorylation was inhibited by the compound. Cell cycle regulation, however, was shown to mediate the expression of multiple genes including metastasis-related genes [13]. Palbociclib has the potential to target these genes to affect breast cancer metastasis. Epithelial-mesenchymal transition (EMT) is a significant process in cancer metastasis. EMT causes cancer cells to gain invasive phenotypes, which has implications for the progression of breast carcinoma to metastasis. Our findings revealed that vimentin and Snail, two EMT-associated proteins, were down-regulated after palbociclib treatment. Furthermore, COX-2, a key oncogene in human breast cancer, was suppressed by the compound. Constitutive COX-2 overexpression is a ubiquitous oncogenic signaling in breast cancer, and COX-2 suppression showed a strong potential for breast cancer treatment. COX-2 expression was elevated in up to 37% of breast cancer cases and correlated with poor prognosis and a high lymphatic metastasis rate [29, 30]. COX-2 induced PGE2 production was reported to regulate breast cancer metastasis through EMT progression [31]. Vimentin and Snail were reduced by COX-2 inhibition [31, 32]. In this study, we found palbociclib inhibited breast cancer cell migration and invasion by targeting COX-2/PGE2. The expression of vimentin and Snail were also decreased, similar to COX-2. Other EMT-related proteins, such as E-cadherin, N-cadherin, MMP-2 and MMP-9, were evaluated but were not clearly detected because of their relatively lower expression levels.

The transcriptional regulation of COX-2 was evaluated in the present study. We found that c-Jun was down-regulated in palbociclib-treated cells. Current evidence indicates that COX-2 transcription activity is regulated by c-Jun, which is the fundamental member and the most potent transcriptional activator of the AP-1 family [22]. Activated AP-1 proteins promote the transcription of their targeted genes. C-Jun was reported to be associated with multiple aspects of progression of malignant disease, including proliferation, apoptosis, invasion and tumorigenesis [33–35]. c-Jun activity is mainly regulated by mitogen-activated protein kinases (MAPKs) pathway. C-Jun N-terminal kinase (JNK) and ERK signaling are two dominating regulatory mechanisms to phosphorylate and stable c-Jun [36, 37]. Activated c-Jun translocates from the cytoplasm to the nucleus to promote COX-2 expression. In this study, we found that *p*-JNK expression was decreased in palbociclib-treated cells in accordance with *p*-*c*-Jun, but there was no reduction in *p*-ERK expression. We further showed that c-Jun bound to the promoter region of COX-2, and its binding ability was weakened after palbociclib treatment. The translocation of c-Jun was also prevented by palbociclib. This result implied that the JNK/c-Jun/COX-2 axis was a vital molecular mechanism in the anti-metastasis activity of palbociclib. NF-kB, another important transcription factor of COX-2, was evaluated in this study but showed no variation.

There are limitations in our study. Our results showed that LPS inducing COX-2 up-regulation could not completely rescue the anti-metastasis activity of palbociclib. This result indicates that c-Jun/COX-2 was an important but not the sole regulatory pathway of palbociclib. Moreover, the crosstalk between the Rb/E2F axis and the c-Jun/COX-2 pathway was not analyzed. C-Jun has the potential to form a polymer with other proteins to promote COX-2 transcription, but the interacting protein of c-Jun has not yet been explored. Moreover, clinical specimen and prognostic information is needed to further support our findings.

In summary, we conclude that palbociclib had a novel anti-metastasis activity in breast cancer cells that was exerted via the c-Jun/COX-2 signaling pathway by inhibiting cell migration and invasion. Palbociclib was believed to be an anti-tumor agent only in ER+ breast cancer in previous studies, our findings noted that both ER+ and ER− breast cancer had the potential to benefit from palbociclib treatment. Furthermore, we revealed that the c-Jun/COX-2 signaling pathway was a promising target of palbociclib. According to our present preclinical data, palbociclib, a small molecule agent, showed enormous potential for breast cancer treatment.

## MATERIALS AND METHODS

### Cell culture and chemicals

MDA-MB-231 cells and T47D cells were obtained from American Type Culture Collection (ATCC, Manassas, VA, USA) and were grown in DMEM (Invitrogen, Carlsbad, CA, USA) supplemented with 10% fetal bovine serum (Invitrogen), 100 U/mL penicillin, and 100 μg/mL streptomycin (Invitrogen). All cells were cultured in a humidified atmosphere of 5% CO_2_ at 37°C. Palbociclib (PD0332991) was a gift from Pfizer (New York, NY, USA) and the COX-2 inhibitor celecoxib was purchased from Sigma (St. Louis, MO, USA). Palbociclib was prepared in dimethyl sulfoxide at a 20 mmol/L solution, and celecoxib was prepared at 100 mmol/L. The stock solutions were stored at −20°C before use.

### Western blot analysis

MDA-MB-231 and T47D cells were plated in 100-mm dishes for 48 hours with different concentrations of Palbociclib (0 μM, 7.5 μM, and 15 μM) or celecoxib (0 μM, 25/50 μM, 50/75 μM). A total of 90 μl of RIPA lysis buffer supplemented with 1% protease Inhibitor (Selleck, Houston, TX, USA) and 1% phosphotransferase inhibitor was used to lyse cells. Approximately 40–80 μg total protein of each sample was separated by electrophoresis and then transferred to a nitrocellulose membrane (Amersham Biosciences, Piscataway, NJ, USA). Rabbit polyclonal antibodies for vimentin, Snail, COX-2, p38, ERK, *p*-ERK, JNK, c-Jun, *p*-c-Jun and histone H3 were obtained from Cell Signaling Pathway Technology (CST, Danvers, MA, USA). Anti-NFκB (p50) antibody was purchased from Santa Cruz Biotechnology (Santa Cruz, CA, USA). Anti-Rb antibody was purchased from Proteintech (Proteintech Group, IL, USA) and anti-*p*-Rb antibody (Ser 780) was obtained from Signalway Antibody (SAB, MD, USA). The secondary antibody was from Promega (Promega Corporation, WI, USA). To detect the protein bands, we used the SuperSignal Chemiluminescent Substrates from Thermo Fisher Scientific.

### Scratch assay

Cells were planted in a 6-well culture plates and incubated overnight to a density of 60%–70%. Cell monolayers were then scratched with a 100-μL yellow pipette tip and washed with PBS three times to remove detached cells. The wounded areas were imaged using an Olympus microscope and marked. The indicated doses of Palbociclib, celecoxib or LPS were applied to culture cells for 48 hours at 37°C in 5% CO_2_. The culture medium was removed, and the same areas were imaged again to observe the wound gap.

### Cell invasion assay

The cell invasion assay was conducted using BD PET-track-etched membrane invasion chamber with 20 μl of diluted Matrigel (BD Biosciences, San Jose, CA, USA). The cells were pretreated with the indicated doses of Palbociclib,celecoxib and LPS for 48 hours and starved for 24 hours. Then, 4 × 10^4^ cells were added to the upper chamber without FBS. The lower chamber was filled with 1 ml of DMEM with 20% FBS. The chambers were incubated at 37°C for 18 hours, and the invaded cells were stained with crystal violet. The cells that adhered to the membrane were imaged and counted.

### PGE2 assay

Cells were seeded in 6-well culture plates and treated with or without Palbociclib or celecoxib in DMEM. The cell culture supernatants were collected after 48 hours and centrifuged. PGE2 levels were determined using a enzyme linked immunosorbent assay (ELISA) kit (R&D System, Minneapolis, MN, USA). The experiments were performed according to the manufacturer's instructions.

### Confocal immunofluorescence assay

MDA-MB-231 cells and T47D cells were cultured in cover slips after exposure to drug for 48 hours. The cells were than fixed with 4% paraformaldehyde for 10 minutes and permeabilized with 0.5% Triton-X for 8 minutes. The cells were blocked with 5% bovine serum albumin (BSA) for 30 min. The primary antibody for c-Jun was diluted with 1% BSA at a density of 1:50 and added to the samples and incubated at 4°C for 12 hours. Then, a 1:200 dilution of rhodamine-conjugated secondary antibodies (Dylight 549) was added to the samples and incubated protected from light for 30 minutes. The cell nuclei were stained with 0.5 μg/ml of 4′,6-diamidino-2-henylindole (DAPI) for 10 minutes. Cell samples were washed three times with PBS between each step. The samples were observed with a OLYMPUS FV1000 confocal microscope.

### DNA-protein binding by streptavidin-agarose pull-down assay

The COX-2 core promoter binding ability of c-Jun was evaluated using a streptavidin-agarose pull-down assay according to our previously described protocol [22]. A 478-bp DNA probe was used in this assay. The double-stranded probe corresponded to the COX-2 promoter sequence (−30 to −508) and is labeled with biotin. The probe was synthesized with a biotin-labeled primer (forward, 5′-ACGTGACTTCCTCGACCCTC-3′, reverse, 5′-AAGACTGAAAACCAAGCCCA-3′) by polymerase chain reaction (PCR).

C-Jun expression was analyzed by Western blotting.

### Stable transfection of luciferase

pMX-Luc2 plasmid was transfected into 293FT cells, and the supernatant was collected after 48 h. The luciferase over-expressing lentivirus was isolated from the supernatant. MDA-MB-231 cells were cultured in 6-well culture plates until the cell density reached approximately 40%–50%. The cells were washed with PBS, and 2 ml of DMEM without FBS was added to each well. A 100-μl suspension of the virus with 5 μg/ml polybrene was used to infect the MDA-MB-231 cells. At 72 hours after infection, the cells were selected with 1.0 μg/ml puromycin, and the selection was repeated twice. The expression level of luciferase in MDA-MB-231 cells was evaluated by Promega GloMax^®^ 20/20 Fluorescent Module.

### Animals and treatment

Female nude mice aged 4–5 weeks were purchased from Guangdong Laboratory Animal Center and quarantined for one week before use. Animal care and experiments involved in this study were consistent with Accreditation of Laboratory Animal Care International guidelines. Animals and protocols were approved by the guidelines established by the Animal Care Committee at Sun Yat-sen University.

MDA-MB-231-LUC cells (5 × 10^5^) were suspended in 100 μl of normal saline (NS) and injected through the tail vein. One week after the injection, the animals were randomly divided into two groups and treated as follows: the treatment group, palbociclib at 200 mg/kg via oral gavage twice a week for 3 weeks [23]; and the control group, which received vehicle (sodium lactate ringer's injection). The weights of the mice in each group were measured weekly.

### Bioluminescence imaging

The bioluminescence imaging was supported by Guangzhou RiboBio Co., LTD. Bruker xtreme *in-vivo* imaging system was used to observe the fluorescence signal in mice. The mice were sedated by isoflurane-inhaled anesthesia for 15 minutes before imaging. The luminescence exposure time was 60 seconds, and the *x*-ray exposure tine was 20 seconds.

### Histopathology

The HE staining, immunohistochemical staining (IHC) and histopathology analysis were supported by Sun yet-son University Cancer Center Pathology Department. The concentration of primary antibody was 1:900 for COX-2 and 1:800 for c-Jun.

### Data analyses

All data and statistic graphs were analyzed using GraphPad Prism 6.0. Student's t test was used to compare the differences between control and treated groups. The mean ±SEM is presented in all graphs, and significance was defined as *p* < 0.05.

## SUPPLEMENTARY FIGURE


