# Supplementary material for: Palbociclib inhibits epithelial-mesenchymal transition and metastasis in breast cancer via c-Jun/COX-2 signaling pathway
Source: Oncotarget. 2015 Oct 19;6(39):41794–808. doi: 10.18632/oncotarget.5993 (PMC4747189; doi:10.18632/oncotarget.5993)
Supplement: Supplementary file 1 [file oncotarget-06-41794-s001.pdf]

SUPPLEMENTARY FIGURE

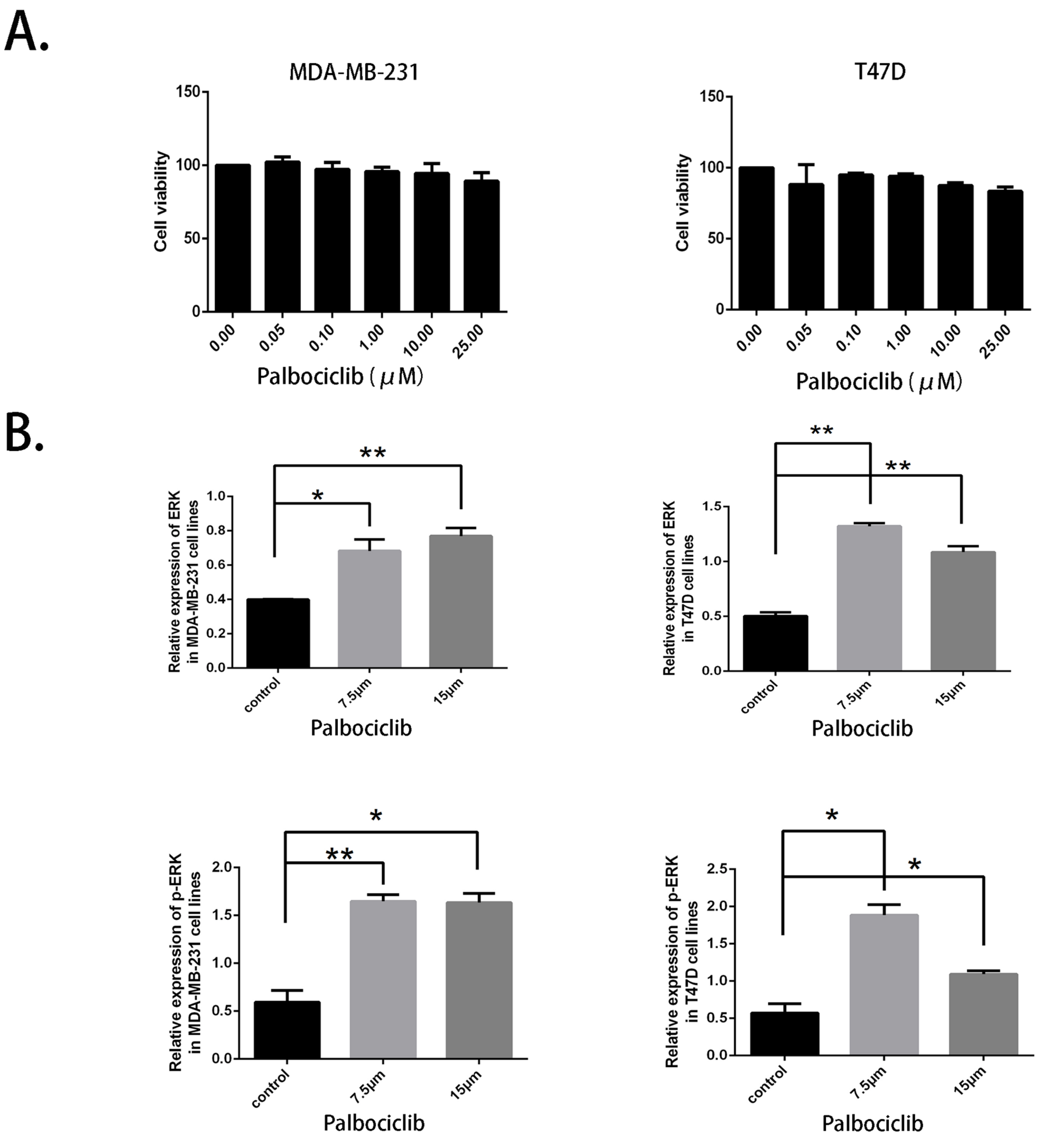

Supplementary Figure S1: A. Palbociclib showed no significant proliferation inhibition activity in MDA-MB-231 and T47D cell lines**. B.** The expression level of ERK and p-ERK was increased after palbociclib treated. The gels of western blot were quantified by gray intensity using IMAGE J.
